# Rare intraoperative findings during the management of pediatric inguinal pathologies: a decade of experience

**DOI:** 10.3389/fped.2025.1643725

**Published:** 2025-11-11

**Authors:** Mostafa Zain, Sameh Shehata, Ahmed Elrouby

**Affiliations:** Pediatric Surgery Department, Faculty of Medicine, Alexandria University, Alexandria, Egypt

**Keywords:** inguinal hernia, ectopic testis, hydrocele, Amyand’s hernia, looping vas deferens, double vas deferens, splenogonadal fusion, ureteringuinal hernia

## Abstract

**Background:**

Pediatric inguinal surgeries, including procedures for inguinal hernias, undescended testes, and hydroceles, are among the most common surgical interventions in children. While these conditions are well-understood, rare intraoperative findings and anatomical variations can complicate surgical management. This study reviews a decade of experience at a single institution to evaluate the incidence, management, and outcomes of such rare findings.

**Methods:**

A retrospective observational study was conducted at a tertiary pediatric surgery center in Egypt, analyzing all inguinal surgeries performed between 2013 and 2022. Patients aged ≤14 years with complete operative records were included. Rare findings were defined as anomalies not typically encountered in standard procedures and were independently reviewed by two surgeons. Data were extracted from surgical logs, operative notes, and electronic records.

**Results:**

Among 8,756 patients (85.5% male, 14.5% female), rare intraoperative findings were identified in 69 cases (0.8%). These included Amyand's hernia (*n* = 12), looping vas deferens (*n* = 34), splenogonadal fusion (*n* = 2), complete androgen insensitivity syndrome (CAIS, *n* = 7), congenital unilateral absence of the vas deferens (CUAVD, *n* = 5), crossed ectopic testes (*n* = 5), ureteroinguinal hernia (*n* = 1), Mayer–Rokitansky–Küster–Hauser (MRKH) syndrome (*n* = 1), and encysted hydrocele (*n* = 1, female). Most anomalies were identified incidentally, with management tailored to preserve function and minimize complications.

**Conclusion:**

Rare findings in pediatric inguinal surgery, though uncommon (<1%), necessitate surgical adaptability and awareness. Preoperative imaging and laparoscopic techniques enhance detection and management. Training programs should emphasize these anomalies to optimize outcomes.

## 1 Introduction

Inguinal surgeries in pediatric patients are among the most commonly performed surgical procedures, primarily addressing conditions such as inguinal hernias, undescended testes (UDT), and hydroceles. Inguinal hernia remains the most prevalent surgical issue encountered by pediatric surgeons, with an incidence in children ranging from 0.8% to 4.4%, comprising more than 15% of the workload in contemporary pediatric surgical practice (1).

The incidence of UDT is reported to affect approximately 1%–3% of full-term male neonates, with a significantly higher prevalence of up to 30% in premature infants. Of these cases, intra-abdominal testis (IAT) is estimated to represent approximately 20% of occurrences (2).

Hydrocele occurs in approximately 1%–2% of full-term neonates and 10%–30% of preterm infants, with 2%–4% of cases necessitating surgical intervention, typically when the hydrocele persists beyond the age of 1–2 years or leads to complications (3).

While the management of these conditions is generally well established, the occurrence of rare intraoperative findings or unusual anatomical variations can pose significant challenges during surgery. These findings may necessitate modifications to the surgical approach and impact the overall management and patient outcomes (4).

Over the past 10 years at our institution, we have encountered several such rare findings during pediatric inguinal surgeries, providing an opportunity to evaluate their clinical implications, management strategies, and surgical outcomes. This study aims to review these uncommon intraoperative findings, discuss their potential effects on surgical management, and offer recommendations for optimizing surgical strategies in future cases.

## 2 Materials and methods

This retrospective observational study was conducted at our institution, a tertiary pediatric surgery center in Egypt, and included all pediatric inguinal surgeries performed between January 2013 and December 2022. The aim was to review the spectrum of rare intraoperative findings encountered during surgery for common pediatric inguinal conditions.

The study enrolled patients within the pediatric age range (up to 14 years old) who underwent inguinal surgery for hernia, hydrocele, or UDT and had complete operative and hospital records. The upper age limit reflects institutional policy, whereby patients older than 14 years are referred to adult surgical services. Patients were excluded if they were operated on for unrelated conditions such as trauma or tumors, had incomplete documentation, or underwent repeat or staged procedures—only the first operation was included for analysis.

Data were collected retrospectively from surgical logs, operative notes, and electronic medical records. Extracted variables included patient demographics (age and sex), clinical presentation, type and approach of the surgical procedure (open or laparoscopic), intraoperative findings, and final diagnosis. All surgeries during the study period were documented using standardized operative templates that were implemented institution-wide in early 2012 to improve data consistency. These templates captured key variables, including patient demographics (age, sex, relevant medical history), preoperative diagnosis and laterality, intraoperative findings, details of the surgical procedure (type of intervention, technique, and approach), operative parameters (duration and intraoperative complications), and immediate postoperative outcomes (early complications, need for re-intervention, and length of hospital stay). This structured approach facilitated consistent reporting and enabled accurate retrospective analysis.

For the purpose of this study, rare findings were defined as any intraoperative anomaly not typically expected during standard pediatric inguinal surgeries. The inclusion of each case as a rare finding was based on established literature describing their low incidence in pediatric inguinal surgery. To ensure the reliability of the findings, all cases with suspected rare intraoperative anomalies were reviewed independently by two experienced pediatric surgeons. In cases of disagreement, a consensus was reached through discussion. Where available, operative photographs and videos were also reviewed to validate the findings.

The study was conducted in accordance with the ethical standards of the institution. Approval was obtained from the institutional review board, and the requirement for informed consent was waived due to the retrospective nature of the study and the anonymization of patient data.

## 3 Results

This retrospective study analyzed the medical records of pediatric patients who underwent inguinal surgeries at our institution over a 10-year period (2013–2023). A total of 8,756 pediatric patients were included, with 7,489 males (85.5%) and 1,267 females (14.5%), yielding a male-to-female ratio of 5.9:1. The age of patients in this cohort ranged from 1 month to 18 years.

The most frequently performed procedures were inguinal hernia repair, accounting for 63% (*n* = 5,511) of cases, followed by orchiopexy for undescended testis (UDT) in 29% (*n* = 2,536) of cases and patent processus vaginalis ligation (PPV) for hydrocele in 8% (*n* = 708) of cases. Surgical approaches were divided between laparoscopic techniques, utilized in 28% (*n* = 2,457) of cases, and open surgery, which constituted the remaining 72% (*n* = 6,299).

Intraoperative findings revealed rare or unexpected conditions in 69 patients (0.8%), with different distribution among different procedures as shown in Tables 1 and 2.

**Table 1:** Surgical procedures and distribution of rare intraoperative findings (*n* = 69).

Surgical procedure	Total (*n*)	Rare findings (*n*)	Types of rare findings
Inguinal hernia repair	5,511	26	Amyand's hernia (12) CAIS (7) CUAVD (5) Crossed ectopic testis (4) MRKH syndrome (1)
Orchiopexy for undescended testis (UDT)	2,536	37	Looping vas deferens (34) Splenogonadal fusion (2) Double vas deferens (1)
Hydrocele	708	1	Encysted hydrocele in a female (1)
Laparoscopic hernia/UDT repair	Included in the above totals	5	Crossed ectopic testis (1) MRKH syndrome (1) Ureteroinguinal hernia (1) Crossed ectopic testis (1)
Total (all procedures)	8,756	69	

**Table 2:** Rare intraoperative findings—incidence, sex distribution, and primary diagnosis.

Rare finding	*n*	Incidence (%)	Sex distribution	Primary diagnosis
Amyand's Hernia	12	0.14	Male	Inguinal hernia
Looping vas deferens	34	0.39	Male	Undescended testis
Splenogonadal fusion	2	0.02	Male	Testicular lump/UDT
CAIS	7	0.08	Phenotypic female, genetically male (46,XY)	Inguinal hernia
Double vas deferens	1	0.01	Male	Undescended testis
CUAVD	5	0.06	Male	Inguinal hernia
Crossed ectopic testes	5	0.06	Male	UDT + contralateral hernia
Ureteroinguinal hernia	1	0.01	Male	Irreducible hernia
MRKH syndrome	1	0.01	Female	Inguinal hernias
Encysted hydrocele (female)	1	0.01	Female	Inguinal swelling

### 3.1 Amyand's hernia

Twelve male patients, aged 3–5 years, were intraoperatively diagnosed with Amyand's hernia during elective right inguinal herniotomy. In all cases, a macroscopically normal appendix was identified, reduced without resection, and standard herniotomy was performed in accordance with the Losanoff and Basson classification (5).

### 3.2 Looping vas deferens

**Figure 1:**
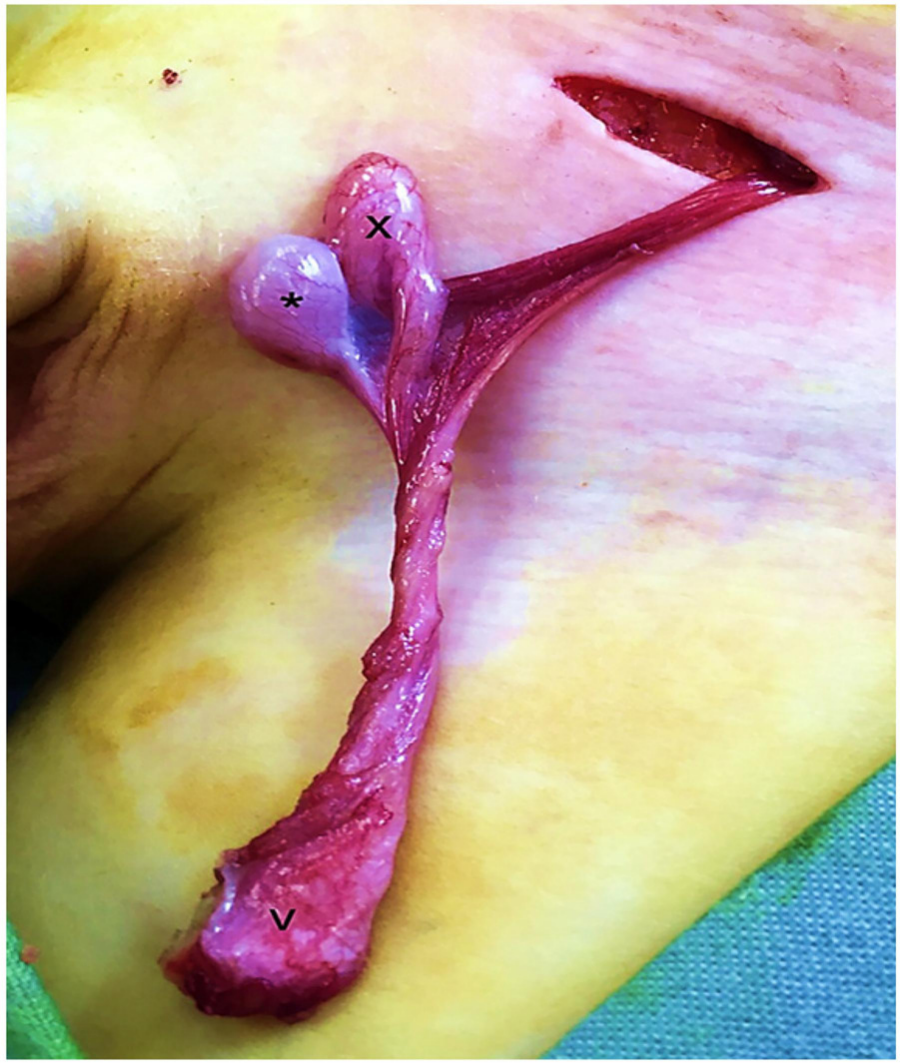
An intraoperative view of one of our cases showing an extra-looping vas deferens (v) leading into the epididymis (x) connected to the testis (*).

A looping vas deferens was observed in 34 patients undergoing orchiopexy—14 through an inguinal approach for palpable testes and 20 via laparoscopy for impalpable testes. The anomaly was identified during gubernacular dissection and managed with meticulous preservation of the vas. All cases demonstrated an associated patent processus vaginalis (Figure 1).

### 3.3 Splenogonadal fusion

**Figure 2:**
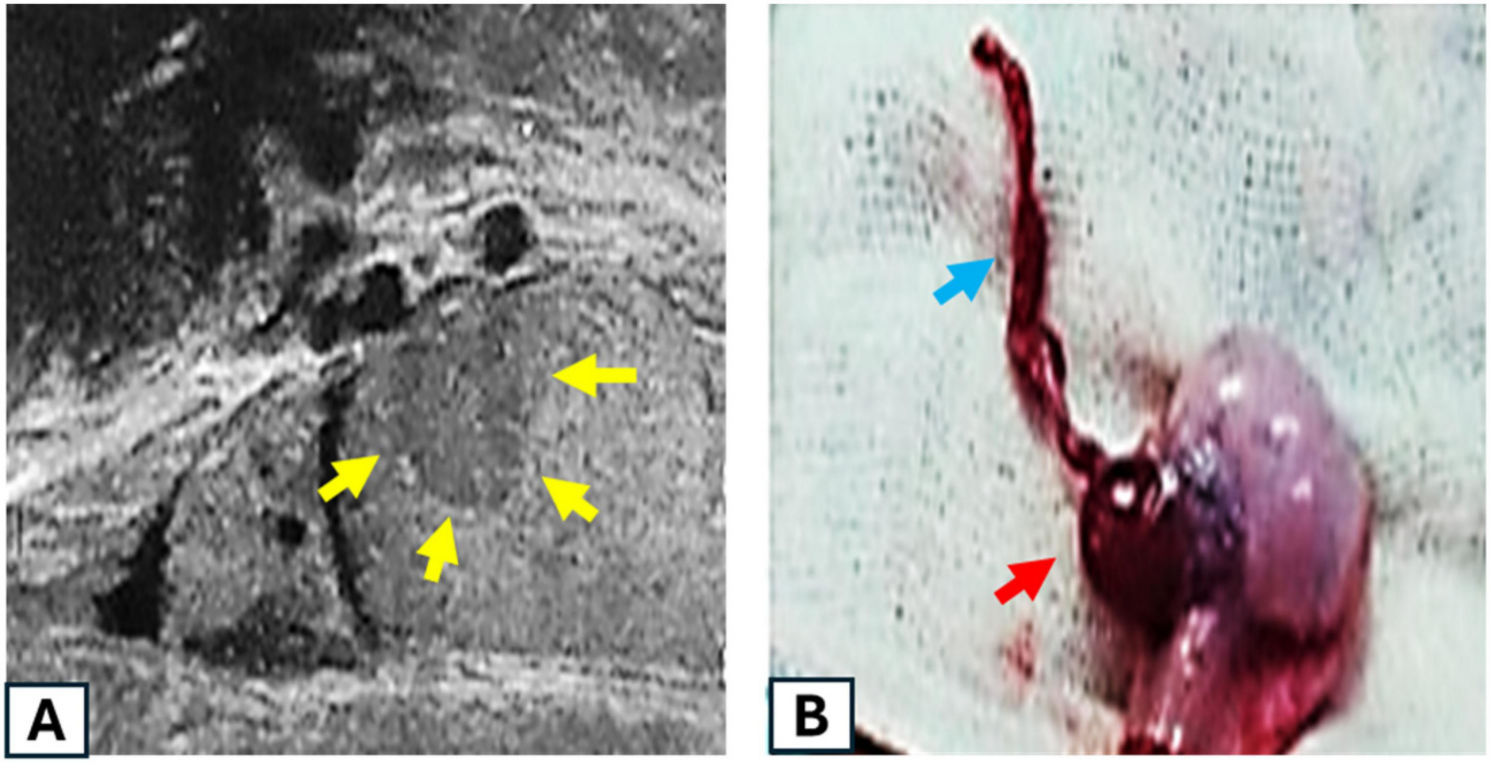
**(A)** First case: scrotal ultrasound revealing a hypervascular, hypoechoic testicular nodule measuring 4 mm × 6 mm × 5 mm (indicated by yellow arrows). **(B)** Second case: intraoperative images showing a mass attached to the head of the epididymis (red arrow) and proximally connected to a cord-like structure (blue arrow).

Splenogonadal fusion (SGF) was diagnosed in two cases. The first case involved a 7-year-old male who presented with left testicular pain and no significant medical history. Physical examination revealed a small, solid nodule at the upper pole of the left testis. Scrotal ultrasonography (US) demonstrated a hypoechoic, hypervascular nodule measuring 4 mm × 6 mm × 5 mm (Figure 2A). Given the high clinical suspicion of a testicular tumor, serum tumor markers, including alpha-fetoprotein, human chorionic gonadotropin (hCG), and lactate dehydrogenase (LDH), were assessed and found to be within normal limits. A testicular-sparing lumpectomy was performed via an inguinal approach, with intraoperative frozen section pathology revealing no evidence of malignancy. Histopathology confirmed ectopic splenic tissue, consistent with discontinuous SGF.

The second case involved a 2-year-old male with a palpable left inguinal UDT. Intraoperatively, a firm, dark mass was attached to the head of the epididymis, proximally connected to a cord-like structure extending into the abdominal cavity through the inguinal canal. The cord-like structure was traced as far as possible from the extended inguinal incision and excised (Figure 2B). A left orchiopexy was performed, with the proximal portion of the fibrous cord left *in situ*. Histopathology confirmed splenic tissue with preserved architecture and congested sinusoids, consistent with continuous SGF.

### 3.4 Complete androgen insensitivity syndrome

Seven phenotypically female patients were diagnosed with complete androgen insensitivity syndrome (CAIS), initially suspected during inguinal herniotomy when a testis-like structure was identified within the hernial sac. The sac was ligated, the gonads were left in their original inguinal position, and the procedures were completed uneventfully. All patients were referred for multidisciplinary evaluation by a pediatric endocrinologist. Postoperative abdominal US revealed absent uterus and ovaries, and karyotyping confirmed a 46,XY genotype in all cases.

### 3.5 Double vas deferens

**Figure 3:**
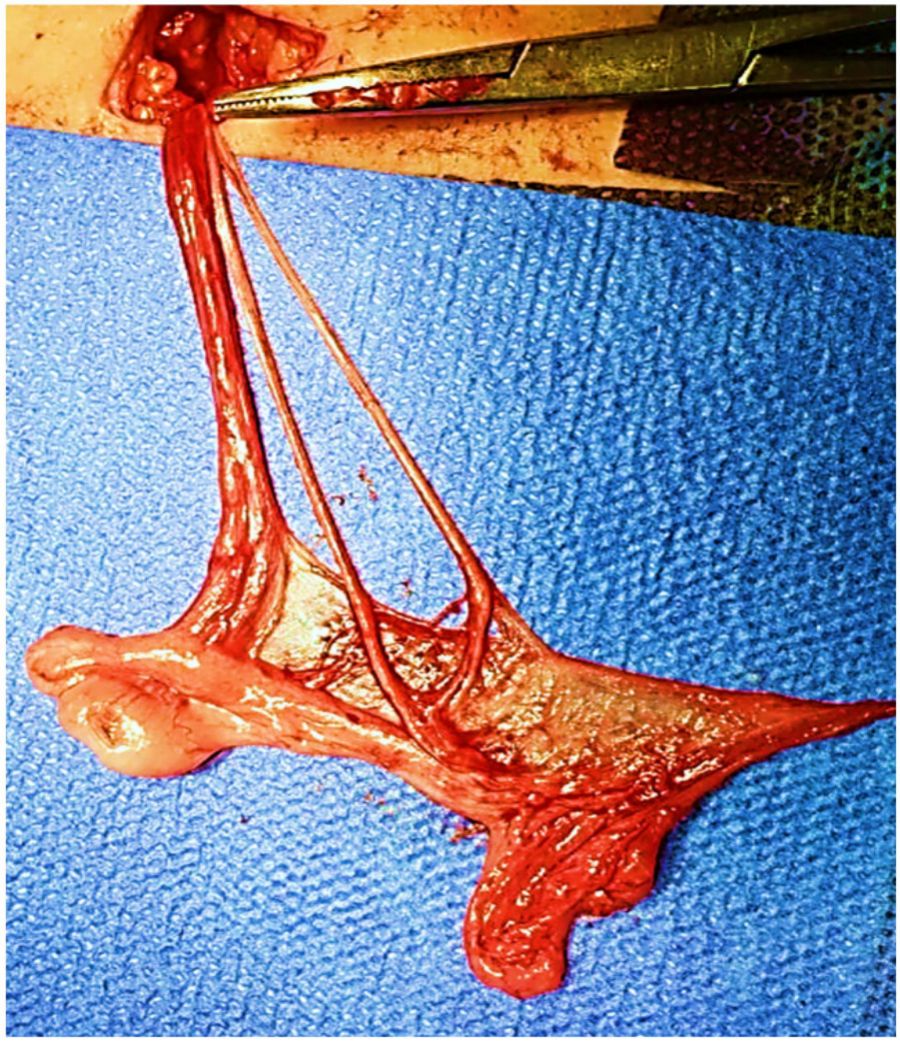
Intraoperative image displaying two vasa deferentia connecting independently to the tail of the epididymis.

A 14-month-old male with a left undescended testis and a history of bilateral talipes equinovarus underwent inguinal orchiopexy. Intraoperatively, two separate vasa deferentia were identified, each independently entering the epididymis (Figure 3). The hernia sac was ligated, and the testis was fixed in a subdartos pouch. Postoperative Doppler US confirmed normal blood flow in both testes, with no waveform detected in the vasa deferentia, as expected for his age. An abdominal ultrasound showed no genitourinary abnormalities.

### 3.6 Congenital unilateral absence of the vas deferens

Congenital unilateral absence of the vas deferens (CUAVD) was incidentally diagnosed in five male infants aged 3, 7, 8, 13, and 20 months. Four presented with a unilateral inguinal hernia, while the 13-month-old had bilateral hernias. In all cases, the absence of the vas deferens was noted intraoperatively on the left side. One patient (8 months old) had a preoperative diagnosis of left renal agenesis. Postoperative abdominal US revealed ipsilateral renal agenesis in three additional patients (aged 3, 7, and 20 months) and an ectopic pelvic kidney in the 13-month-old. The contralateral testis and vas deferens were sonographically normal in all cases. All patients were referred for genetic counseling and evaluation for possible cystic fibrosis transmembrane conductance regulator (CFTR) gene mutations.

### 3.7 Crossed testicular ectopia

**Figure 4:**
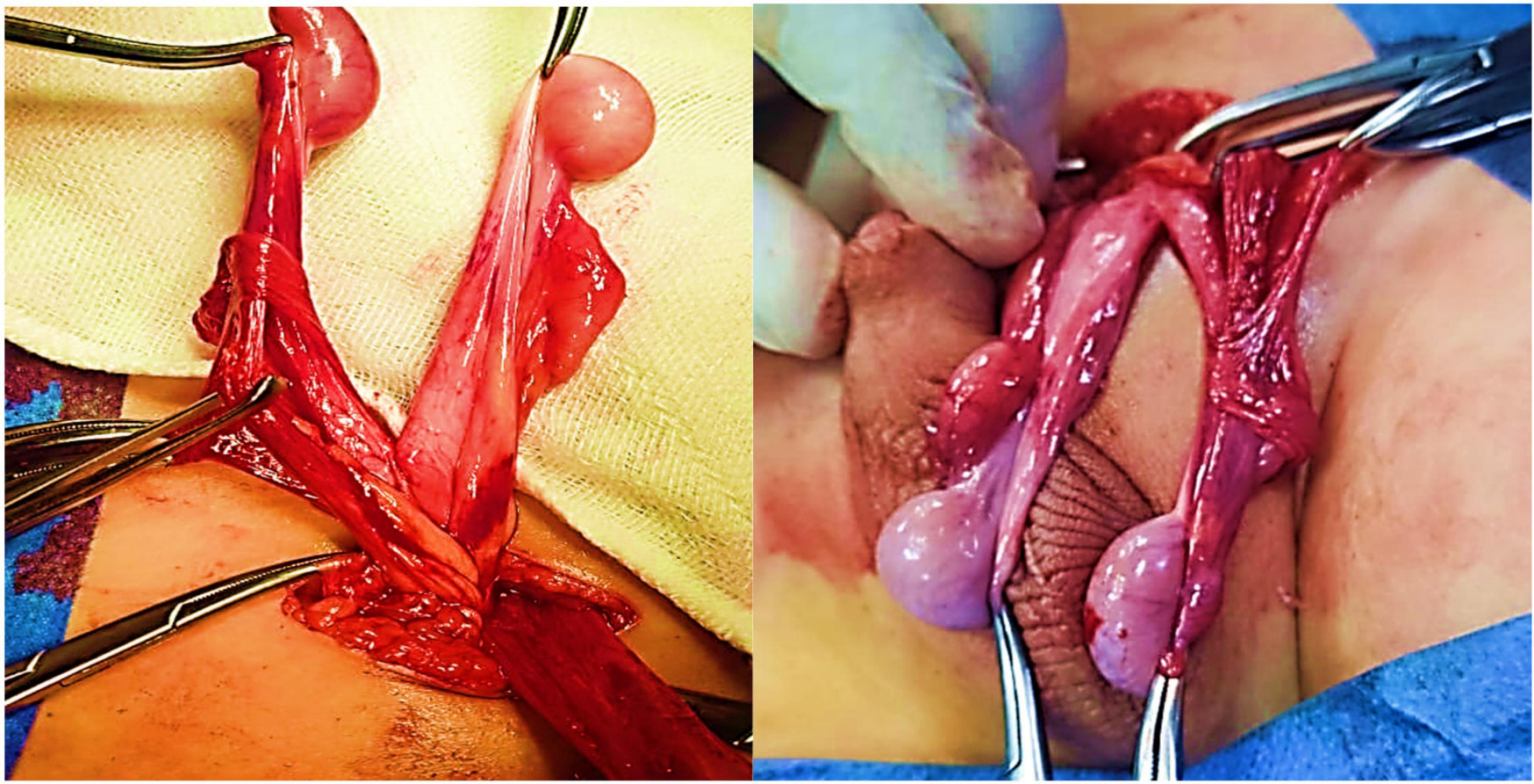
An intraoperative view of a case with crossed testicular ectopia.

**Figure 5:**
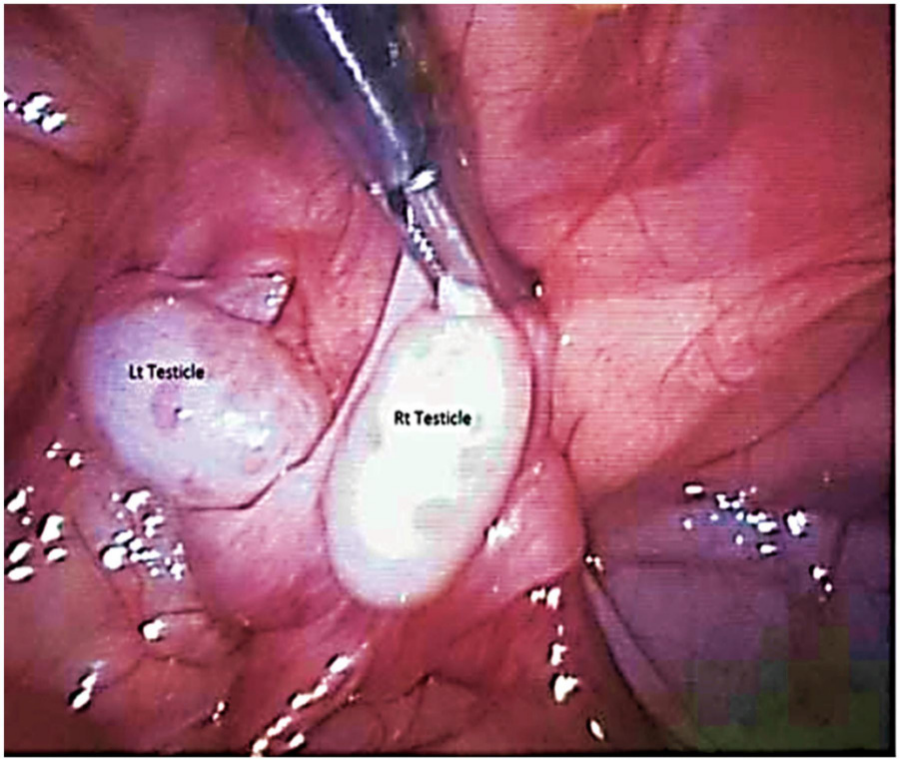
Laparoscopic view of a case of crossed testicular ectopia in a patient with bilateral impalpable testes.

Five patients were diagnosed with crossed testicular ectopia. In four patients, the diagnosis was made intraoperatively during unilateral inguinal herniotomy for a clinically detectable hernia, with a contralateral impalpable testis. Herniotomy was completed, and transseptal orchiopexy was performed in all four patients (Figure 4). In the fifth patient, diagnostic laparoscopy performed for bilateral impalpable testes demonstrated both gonads situated intra-abdominally on the right side, adjacent to the right deep inguinal ring (Figure 5). The vas deferens and vessels of the ectopic left testis were observed crossing the midline posterior to the bladder neck and coursing in close association with the right vas deferens. No Müllerian duct remnants were detected. Following meticulous peritoneal dissection, both testes were adequately mobilized while preserving their vascular supply. The gonads were then delivered through a right inguinal incision, and bilateral transseptal orchiopexy was accomplished in a single stage, securing each testis in a hemiscrotum.

### 3.8 Ureteroinguinal hernia

**Figure 6:**
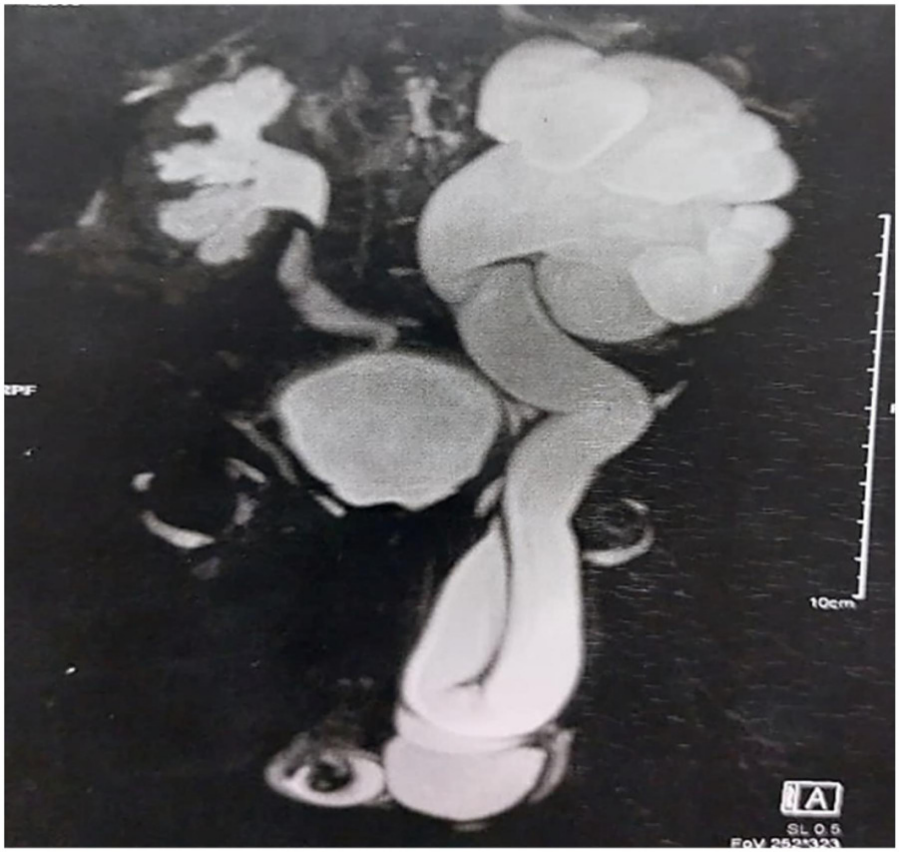
Preoperative MRU demonstrating severe left-sided hydroureteronephrosis with dilated, scattered calyces and multiple ureteral kinks. The dilated ureter extends into a moderate-sized, fat-containing inguinal hernia.

A 3-month-old male infant presented with a 2-day history of irreducible left inguinal swelling without signs of intestinal obstruction. US revealed Grade 4 left hydronephrosis with a dilated ureter traversing the inguinal canal. Magnetic resonance urography (MRU) confirmed marked left-sided hydroureteronephrosis with a tortuous ureter extending into a fat-containing inguinal hernia (Figure 6). Voiding cystourethrogram excluded vesicoureteral reflux, and a diagnosis of primary obstructing megaureter with ureteroinguinal hernia was established. A dimercaptosuccinic acid (DMSA) scan showed decreased left renal function (26%) without evidence of scarring. The patient underwent laparoscopic hernia repair with ureterostomy: The hernial sac was dissected, the ureter reduced, and the internal ring repaired. The dilated ureter was partially excised and exteriorized as a ureterostomy, with ureteric reimplantation planned at 1 year of age.

### 3.9 Mayer–Rokitansky–Küster–Hauser syndrome

A 14-month-old girl with a bilateral canal of Nuck hernias underwent laparoscopic repair. Intraoperatively, the uterus and fallopian tubes were absent, with normal-appearing ovaries. External examination showed the presence of only a urethral opening within the vestibule, with no vaginal opening. Postoperative imaging confirmed uterine and vaginal agenesis, normal ovaries, a left pelvic kidney, and absent right kidney—findings consistent with atypical Mayer–Rokitansky–Küster–Hauser (MRKH) syndrome.

### 3.10 Encysted hydrocele in a female patient

**Figure 7:**
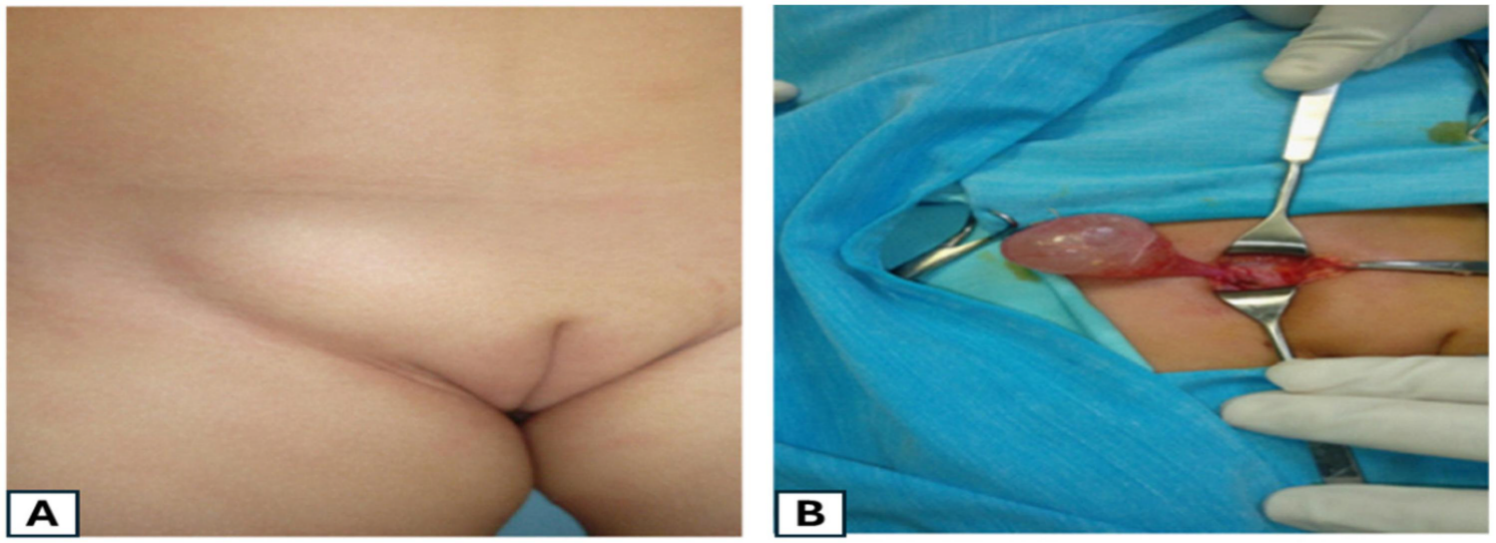
**(A)** A female patient presented with right inguinal swelling. **(B)** Operative view of the right female hydrocele. Reproduced from (6), licensed under CC BY.

A 3-year-old girl presented with a year-long history of a gradually enlarging right, irreducible inguinal swelling. Examination suggested a hydrocele with positive fluctuation and transillumination tests, confirmed by US as a cystic lesion with septations and normal internal genitalia. Surgical exploration revealed a cystic fluid-filled mass (Figure 7). Hydrocelectomy with ligation of the communication was performed.

## 4 Discussion

Pediatric inguinal surgeries are among the most commonly performed surgical procedures in the pediatric population. While these procedures are generally considered safe, they present unique challenges due to the delicate anatomy of pediatric patients. A further challenge emerges when rare or unexpected intraoperative findings are encountered, requiring careful evaluation and precise surgical decision-making to ensure optimal outcomes and minimize complications. Unusual findings during pediatric inguinal surgery can range from the presence of unexpected organs within the hernia sac to anatomical variations that require specialized management. For example, the management of the appendix in Amyand's hernia differs according to its status, and crossed testicular ectopia requires careful management to avoid postoperative atrophy (7).

The incidence and types of rare findings in our study are consistent with those reported in the literature. For example, Amyand's hernia, SGF, and CAIS have been described in similar proportions in other studies (7). However, the relatively high incidence of looping vas deferens in our series (0.39%) compared with previous reports may reflect differences in surgical technique or diagnostic criteria. Additionally, the identification of extremely rare conditions such as ureteroinguinal hernia and MRKH syndrome underscores the value of large-scale retrospective studies in documenting rare surgical findings (8).

Pediatric inguinal herniotomy, being the most common operation in the pediatric age group, requires that the pediatric surgeon take care of any unusual findings regarding its content to avoid complications and make the right decision regarding the management of such situations. Advances in ultrasound examination of the inguinal hernia have increased the preoperative diagnosis of rare findings of hernia contents to avoid intraoperative surprises. Moreover, the addition of laparoscopy in the management of pediatric inguinal hernia has helped a lot in the diagnosis of such situations (9).

Amyand's hernia is an uncommon finding in pediatric inguinal hernia (1%), with the presence of the appendix as the content of its sac. A wide range of presentations of such situations was observed from incidental findings during herniotomy without any signs or symptoms to well-established appendicitis with epigastric or periumbilical pain and minimal localization to the right iliac fossa, together with compressible swelling in the inguinal or inguinoscrotal region, which can also progress to perforation and abscess formation. Management of such a situation differs according to the condition of the appendix, and routine appendectomy is not mandatory in all cases, so careful examination of the appendix must be considered if it is encountered in the contents of an inguinal hernia (10, 11).

The association of vasoepididymal anomalies, isolated epididymal anomalies, and testicular epididymal junction anomalies with cryptorchidism is found in approximately 32%–72% of patients, being more common than isolated vasal anomalies, which may present as absent vas, vasal duplication, and long-looping vas (8).

The association of long-looping vas with cryptorchidism occurs in approximately 20% of cases in which the testis is found in the inguinal canal or just proximal to it. It is so important to examine the structures distal to the testis in the gubernaculum before its dissection or transection, especially while performing laparoscopy in case of IAT by gently pulling the gubernaculum into the abdomen through the deep inguinal ring or even by performing a small inguinal incision to dissect it from its inguinal attachments. Avoiding inadvertent vasal transfixion is crucial while operating for cryptorchidism to protect against testicular atrophy in the future due to vascular compromise, as the testis receives its dual blood supply from the testicular artery and the artery of the vas (12).

SGF is a rare benign congenital anomaly, primarily affecting males at a male-to-female ratio of 16:1. SGF is classified into two distinct forms: continuous, where the normal, orthotopic spleen is directly connected to the gonad, accounting for 55% of cases, and discontinuous, where ectopic splenic tissue is fused to the gonad without any connection to the normal, orthotopic spleen, accounting for 45% of cases. SGF is most often identified during childhood, incidentally, during surgical procedures for inguinal or testicular abnormalities. While the condition is generally asymptomatic, some cases present with a scrotal mass or symptoms associated with splenic tissue, such as leukemia, mononucleosis, or rupture (13). Despite its benign nature, SGF is often misdiagnosed as a testicular tumor, leading to unnecessary orchiectomy. Improved preoperative evaluation and awareness of SGF are critical to prevent invasive interventions and enhance clinical outcomes (14).

CAIS is a condition in which individuals with a 46,XY karyotype are resistant to androgens due to mutations in the androgen receptor gene, resulting in a female external phenotype despite the presence of testes. In patients with CAIS, the testes are typically undescended and may be located in the inguinal canal or abdomen. This ectopic testicular position often presents clinically as an inguinal hernia, particularly in phenotypic females during infancy or childhood. In fact, the incidental discovery of intra-abdominal or inguinal testes during hernia repair may lead to the diagnosis of CAIS. Therefore, the presence of bilateral inguinal hernias in a phenotypic female child should raise clinical suspicion for underlying disorders of sex development, including CAIS, prompting further evaluation through genetic and hormonal studies (15).

Duplication of the vas deferens is a rare and often underreported anomaly, with only 50 cases documented in the literature. A classification by Liang et al. (16) identifies three types: Type I (duplication within the spermatic cord without polyorchidism), Type II (multiple vasa deferentia with polyorchidism), and Type III (false poly-vasa deferentia with a ureter draining into the ejaculatory system); the current case is classified as Type I. In all the reported cases, duplication was discovered incidentally during surgery. Recognizing the anomaly during surgery is essential to prevent complications such as overlooking the vas deferens during vasectomy, which could result in failed sterilization, or causing injury that may lead to sperm granulomas, chronic pain, or fertility problems.

CUAVD is a rare condition, with an estimated incidence of 0.5%–1% in the male population, though this may be underestimated as it is often diagnosed incidentally during procedures such as inguinal surgery, vasectomy, or during evaluation for infertility. Its prevalence is higher on the left side, occurring twice as frequently as on the right (17). All our cases involved the left side. Ipsilateral renal agenesis is present in approximately 74%–79% of men with CUAVD, with one-third of these individuals also demonstrating abnormalities in the contralateral solitary kidney. Other associated renal anomalies include malrotation, multicystic kidney, ectopic kidney, and horseshoe kidney (17). In our series, four patients (80%) had ipsilateral renal agenesis, and one (20%) had ipsilateral ectopic pelvic kidney.

The two clinical forms of CUAVD, characterized by the presence or absence of renal anomalies, are believed to arise from distinct developmental mechanisms. In cases associated with CFTR gene mutations, vasal agenesis is thought to result from damage to the vas deferens after the separation of the urinary and genital tract components during embryogenesis, leading to secondary atresia of a previously formed vas. In contrast, cases presenting with concomitant renal agenesis are likely attributable to a primary defect in the Wolffian duct, resulting in complete developmental failure of the duct and its derivatives (18). In our series, patients were referred for genetic evaluation at an external institution, although follow-up information was not available. In retrospect, the referral of CUAVD patients for genetic evaluation in our series was not fully justified, particularly in those with unilateral renal agenesis, where the embryologic basis for vasal absence is well established. A more rigorous and stepwise diagnostic strategy would begin with renal US as the initial, non-invasive, and cost-effective investigation. CFTR mutation analysis should be reserved for cases without renal anomalies or for those presenting with bilateral vasal agenesis, especially in regions with a low prevalence of cystic fibrosis.

Crossed ectopic testis could be defined as the migration together with the descent of both testes through a single inguinal canal. The testes may descend to lie in a single hemiscrotum, in the inguinal canal, or even intra-abdominal, and hence the presentation is of bilateral impalpable maldescended testis (19). This anomaly is mostly diagnosed intraoperative while operating on a unilateral inguinal hernia with a maldescended contralateral side and according to this inguinal hernia the condition could be classified into Type 1, in which there is only an inguinal hernia (50%); Type 2, in which there is an associated Mullerian duct structure (30%); and Type 3, in which there is another associated anomaly as hypospadias, scrotal abnormalities, and other urogenital malformations (20%) (20).

The management of crossed ectopic testis is primarily performed via transseptal orchiopexy when the spermatic cord length is sufficient, as is the case in most patients. In situations where the cord is inadequate to achieve tension-free fixation, extraperitoneal testicular transposition may be indicated. This procedure entails an inguinal incision, followed by incision of the floor of the inguinal canal and opening of the fascia transversalis to gain access to the retroperitoneal space. Such exposure allows proximal mobilization of the spermatic vessels, straightening of the cord, and creation of a pathway to transpose the testis to the contralateral hemiscrotum corresponding to its anatomical side. The testis is then delivered through this extraperitoneal route and secured within a dartos pouch without tension, thereby avoiding entry into the peritoneal cavity and minimizing adhesion-related complications. In rare circumstances in which the spermatic cord is exceedingly short and the testis cannot be positioned in a palpable location without tension, orchiectomy may be considered to reduce the risk of future malignancy. Alternatively, a testicular vessel elongation maneuver, such as the Shehata technique, may be employed to facilitate orchiopexy (21, 22).

Ureteroinguinal hernia is an uncommon condition, with fewer than 150 cases documented in the English medical literature. This condition is categorized into two types: extraperitoneal (20%) and paraperitoneal (80%), depending on whether a hernial sac is present. The extraperitoneal type, often linked to embryological anomalies, involves the ureter and retroperitoneal fat herniating into the inguinal canal without a hernial sac. Conversely, the paraperitoneal type, frequently associated with ureteral dilation caused by conditions such as vesicoureteral reflux, posterior urethral valves, or obstructive megaureters, is characterized by the ureter being incorporated into the wall of the hernial sac. Timely preoperative diagnosis is crucial to avoid ureteral injury during surgical repair, though many cases are only identified intraoperatively (23).

MRKH syndrome is a rare congenital disorder, affecting approximately 1 in 4,000–5,000 females, characterized by the partial or complete absence of the uterus and vagina. It is the second most common cause of primary amenorrhea and is frequently associated with renal and skeletal anomalies, such as renal agenesis or ectopic kidneys. The association between MRKH syndrome and inguinal hernias is rare and not well characterized in the literature, with no large-scale epidemiological studies quantifying the exact incidence. The presence of an inguinal hernia in a phenotypic female, particularly if bilateral or accompanied by atypical genital findings, should prompt thorough evaluation for possible underlying congenital anomalies. In this context, careful examination of the external genitalia is essential and may reveal subtle abnormalities, such as the absence of a vaginal opening, that warrant further investigation. Laparoscopy offers both diagnostic and therapeutic advantages: It enables safe and effective hernia repair while providing direct visualization of internal anatomy, which can facilitate the early diagnosis of anomalies (13).

Female hydrocele, resulting from the failure of obliteration of the canal of Nuck—first described by the Dutch anatomist Anton Nuck in 1691 (24)—is a rare condition, accounting for approximately 1% of inguinal anomalies. It is classified into three anatomical types: Type I, the most common, presents as an encysted hydrocele along the course of the round ligament; Type II involves persistent communication with the peritoneal cavity; and Type III consists of bilocular cysts separated by the deep inguinal ring, typically associated with an inguinal hernia (25). Definitive diagnosis is typically established intraoperatively, emphasizing the need for careful dissection and identification of any peritoneal communication to prevent recurrence and avoid injury to adjacent structures (6).

In summary, this large-scale retrospective analysis demonstrates that while rare intraoperative findings occur in less than 1% of pediatric inguinal surgeries, their identification and proper management are crucial for optimal outcomes. Our findings reinforce that these anomalies are not merely academic curiosities but real clinical scenarios that demand surgical adaptability. The study highlights how modern diagnostic techniques, and minimally invasive approaches have transformed our ability to anticipate and address these challenges.

### Study limitations

The primary limitations of this study include its retrospective design and reliance on medical records. Additionally, the rarity of certain conditions under investigation restricts the ability to perform robust statistical analyses, limiting the generalizability of the findings and the capacity to draw definitive conclusions regarding optimal management strategies. Moreover, the absence of long-term follow-up, particularly for patients with CAIS and MRKH syndrome, precludes assessment of delayed outcomes, pubertal development, and potential late complications. Future prospective studies with larger cohorts and extended follow-up are warranted to enhance the reliability and applicability of these results.

## 5 Conclusion

Although pediatric inguinal surgeries are commonly performed, this 10-year review demonstrates that approximately 0.8% of cases involve rare yet clinically significant findings. These anomalies pose diagnostic and therapeutic challenges that may require modifications to conventional surgical approaches. The capacity to accurately identify and effectively manage such unusual findings is essential to minimize the risk of complications. Our results emphasize the importance of comprehensive preoperative evaluation, including the use of imaging modalities when appropriate, as well as the necessity for intraoperative flexibility to optimize patient outcomes.

Moreover, the recognition of these rare entities highlights the need to incorporate their diagnosis and management into pediatric surgical training programs. While the findings from this single-center experience provide valuable insights, multicenter prospective studies are needed to establish standardized guidelines for the management of these uncommon conditions.

## Data Availability

The raw data supporting the conclusions of this article will be made available by the authors, without undue reservation.
